# Effectiveness of Topical Sucralfate in the Management of Diabetic Foot Ulcers: An Open-Labeled Randomized Study

**DOI:** 10.7759/cureus.37570

**Published:** 2023-04-14

**Authors:** Neha Chatterjee, Nishith M Ekka, Mayank Mahajan, Binay Kumar, Nabu Kumar, Arquam Zia, Aravind Devarajan, Archana D Kujur, Dipendra K Sinha

**Affiliations:** 1 General Surgery, Rajendra Institute of Medical Sciences, Ranchi, IND; 2 Surgery, Rajendra Institute of Medical Sciences, Ranchi, IND; 3 Medicine, Rajendra Institute of Medical Sciences, Ranchi, IND; 4 Pharmacology and Therapeutics, Rajendra Institute of Medical Sciences, Ranchi, IND

**Keywords:** topical, diabetes, diabetic foot ulcer, mupirocin, sucralfate

## Abstract

Introduction: Diabetic foot ulcer (DFU) is a major cause of lower limb amputations. Many treatment recommendations have been proposed. This study was conducted to evaluate the effectiveness of topical sucralfate when combined with mupirocin ointment, in the treatment of diabetic foot ulcer in comparison to topical mupirocin alone, in terms of healing rates.

Methods: This open-labeled randomized study was conducted on 108 patients to evaluate the effectiveness of topical sucralfate and mupirocin combination, compared to topical mupirocin alone. The patients were administered the same parenteral antibiotic, and wounds were subjected to daily dressing. The healing rates (determined by the percentage reduction in wound area) in the two groups were calculated. The mean healing rates in both groups were expressed in percentage and compared using the Student's t-test.

Results: A total of 108 patients were included in the study. Male-to-female ratio was 3:1. The incidence of diabetic foot was the highest (50.9%) in the age group of 50-59 years. The mean age of the study population was 51 years. The incidence of diabetic foot ulcers was highest in the months of July-August (42%). A total of 71.2% patients had random blood sugar levels between 150-200 mg/dL, and 72.2% patients had diabetes for five to 10 years. The mean±standard deviation (SD) of the healing rates in the sucralfate and mupirocin combination group and the control group were 16.2±7.3% and 14.5±6.6%, respectively. Comparison of the means by Student's t-test failed to show a statistical difference in healing rates between the two groups (p=0.201).

Conclusion: We concluded that the addition of topical sucralfate does not show any obvious benefits in terms of healing rates in diabetic foot ulcers as compared to mupirocin alone.

## Introduction

The prevalence of diabetes in the age group of 20-75 years is around 10.5% in the global population [1]. Diabetic foot ulcers, which fail to heal are a major cause of lower limb amputations [2]. Development of diabetic foot ulcer results from a combination of multiple factors, out of which peripheral neuropathy is the most important [3]. Peripheral neuropathy may involve motor nerves, sensory nerves, and autonomic nerves. Imbalance between lower limb flexion and extension due to damage of motor innervation results in deformity and change of pressure points, resulting in skin damage and eventually ulcers. The involvement of sensory nerves causes decreased responsiveness to obnoxious stimuli, while autonomic neuropathy leads to dry fissured skin of the foot, thus making it susceptible to injury. A total of 85% of these ulcerations subsequently deteriorate into severe gangrene or infection [3]. The treatment protocol includes clinical assessment of the wound, debridement, management of infection, and off-loading of the ulcer [4,5]. At present, local treatment of diabetic foot ulcers includes topical antimicrobials, such as mupirocin, neomycin, and gentamycin, along with debridement and dressing of the wound [6].

Sucralfate is an aluminum hydroxide salt of the disaccharide sucrose octa sulfate. It is used as an oral medication for treatment of gastrointestinal ulcers and it has revealed potential utility in healing skin wounds [7]. Sucralfate helps in increasing growth factors, especially fibroblast growth factor (FGF) which plays an important role in angiogenesis, and proliferation of dermal fibroblasts and keratinocytes. It also increases interleukin-1 (IL-1) and interleukin-6 (IL-6) secretion, promotes granulation tissue formation, and ultimately promotes ulcer healing [8,9].

We conducted this open-labeled randomized study to determine whether the addition of topical sucralfate in the treatment of diabetic foot ulcers improves the wound healing rate significantly, in comparison to those who receive only topical mupirocin.

## Materials and methods

**Figure 1:**
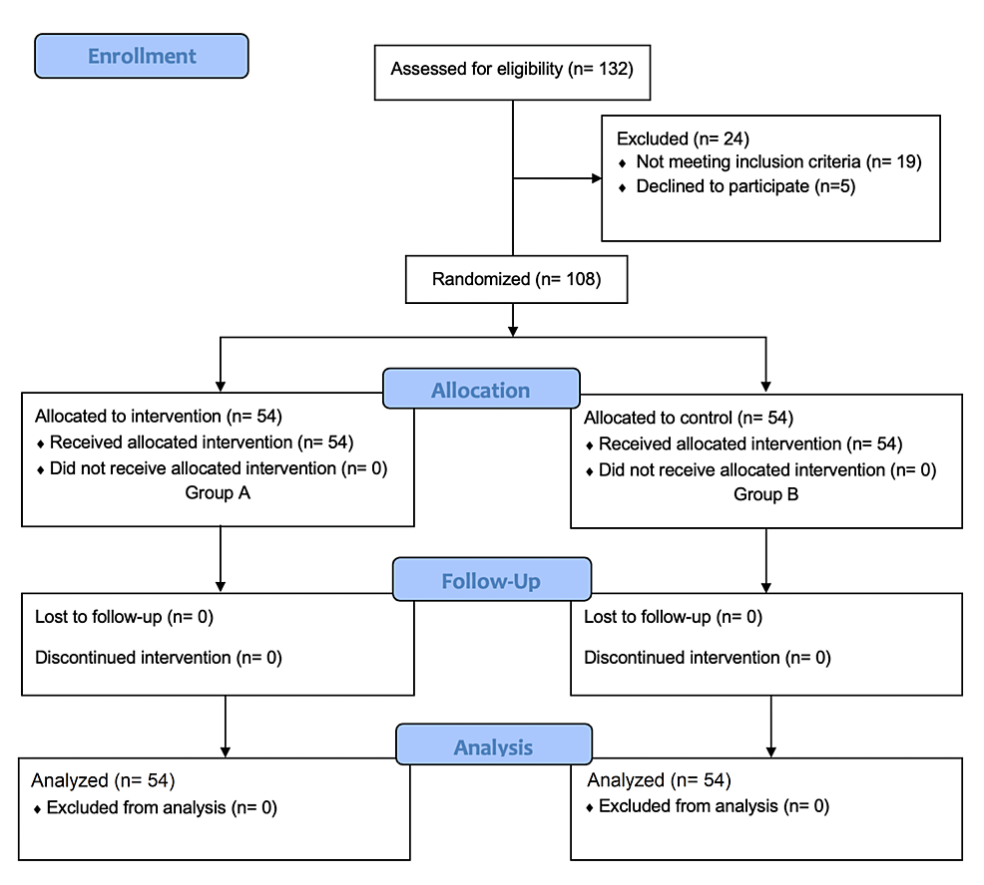
CONSORT flow diagram showing patient inclusion. CONSORT: Consolidated Standards of Reporting Trials

An open-labeled randomized study was conducted from May 2021 to October 2022 in the Department of General Surgery, Rajendra Institute of Medical Sciences, Ranchi, after approval by the Institutional Ethics Committee (#226, dated: 10/05/2021). The sample size was calculated using the following formula: n = Z^2 ^× p (1-p)/d^2^, where n = sample size, Z = 1.96 (for the level of confidence of 95%), p = expected prevalence, and d = precision (0.05) [10]. Initially, 132 patients were assessed for eligibility out of which 24 patients were excluded leaving 108 patients for randomization. These 108 patients, who satisfied the inclusion and exclusion criteria were included in this study. The eligible patients were then allocated either an intervention group that received topical sucralfate and mupirocin combination ointment (group A), or a control group that received topical mupirocin ointment alone (group B). These two groups were then analyzed (Figure 1).

Opaque-sealed envelopes were used to randomize patients to ensure allocation concealment. Informed consent was taken from all the patients included in the study. Any other topical antibiotic, other than mupirocin, was not used. The initial wound area was measured by multiplying length and breadth and expressed in cm^2^, after sharp debridement and development of granulation tissue. Both groups were subjected to daily wound dressing using non-adhesive sterile dressing. In the intervention group, an ointment containing sucralfate in a concentration of 7% IP weight by weight (w/w) and mupirocin in a concentration of 2% IP w/w in a 5 g tube was applied as per the wound area. In the control group, topical mupirocin ointment of concentration 2% IP w/w was used for dressing. The patients were followed up daily for four weeks in both groups. The dressings were changed daily in both groups in the follow-up period of 28 days. The wound area was observed on the 28th day using a transparent graph sheet.

Inclusion and exclusion criteria

Patients were included based on the following criteria: (1) patients with a healing diabetic foot ulcer, (2) ulcer less than 10×10 cm, (3) adult patients between the age of 18-70 years, (4) patients being treated at the Department of General Surgery, Rajendra Institute of Medical Sciences, and (5) patients being treated from March 2021 till the desired sample size is reached. Patients with uncontrolled diabetes and any other comorbidity that affects wound healing were excluded from the study.

Statistical analysis

The data were entered in Microsoft Excel spreadsheet and analysis was done using MedCalc version 14.8.1 (Oostende, Belgium: MedCalc Software Ltd.). The outcome of the study was assessed by average reduction in the ulcer area. The percentage reduction in wound area was recorded for each group and mean±SD was calculated. Continuous variables were compared using Student's t-test. Patients were subdivided into the following four age groups: 30-39 years, 40-49 years, 50-59 years, and 60-69 years. Analysis of variance (ANOVA) was used to analyze the healing rates in various age groups. A p-value of <0.05 was considered statistically significant.

## Results

A total of 108 patients were included in the study which were randomized into two groups: group A and group B. Group A received topical sucralfate and mupirocin combination ointment, while group B received topical mupirocin ointment alone. In this study, we observed a male predominance in number with a male-to-female ratio of around 3:1 (Table 1).

**Table 1:** Gender distribution of study population.

Sex	Number of patients	Percentage (%)
Male	83	77
Female	25	23

**Figure 2:**
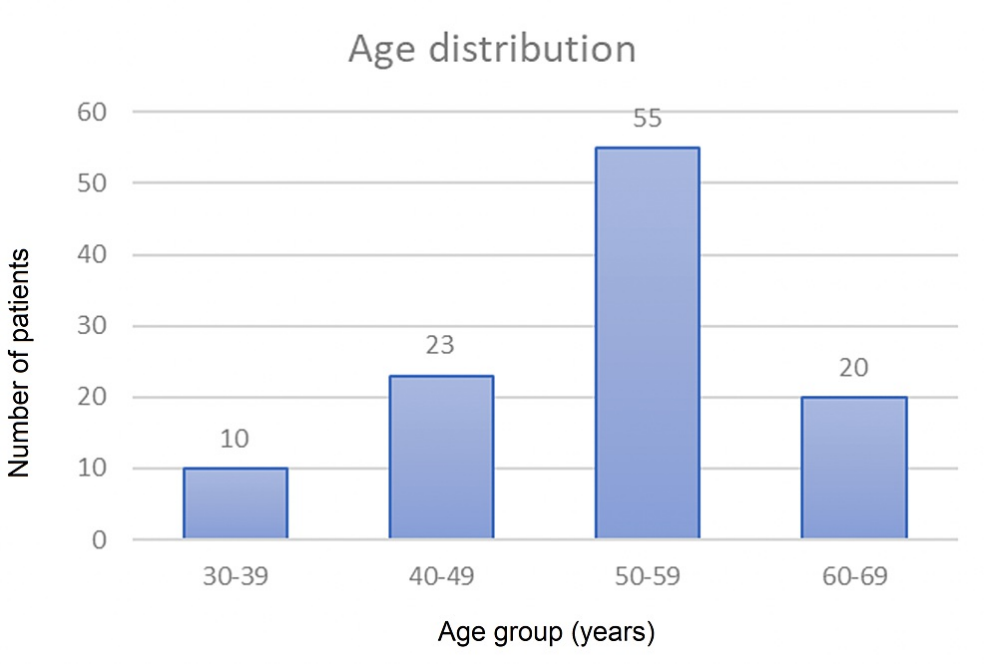
Bar graph showing age distribution of study population.

**Figure 3:**
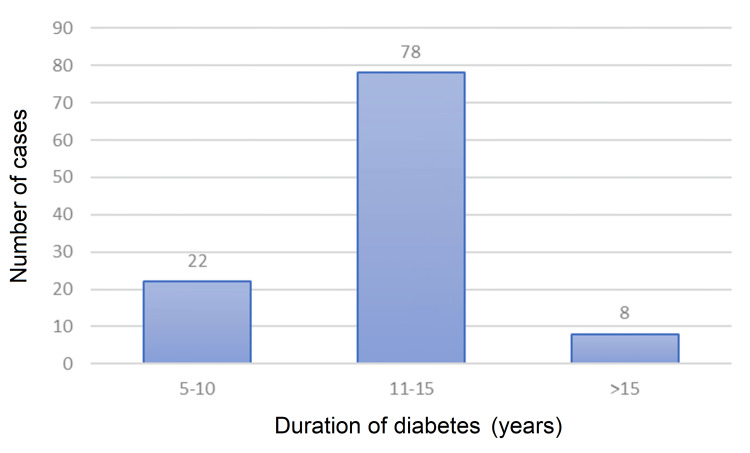
Bar graph showing duration of diabetes in study population.

The mean age at presentation was 51 years in the total population and majority of the patients (50.9%, n=55) belonged to the age group of 50-59 years (Figure 2). Majority of the patients (72.2%, n=78) had diabetes for five to 10 years (Figure 3).

**Figure 4:**
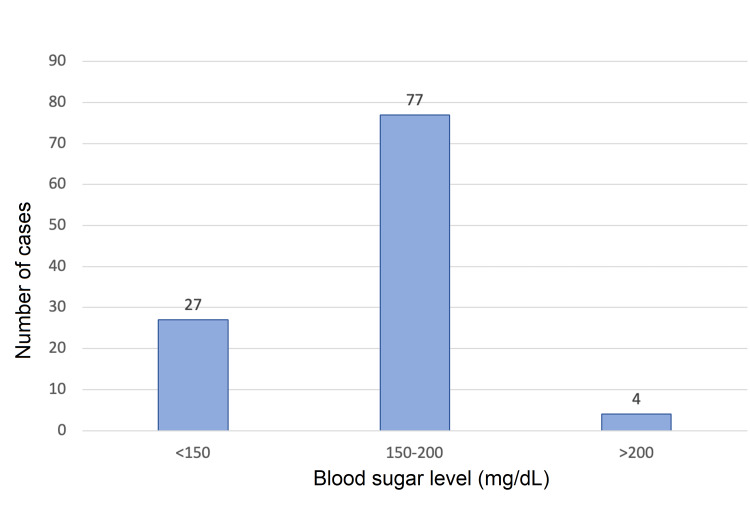
Bar graph showing random blood sugar levels of study population.

**Figure 5:**
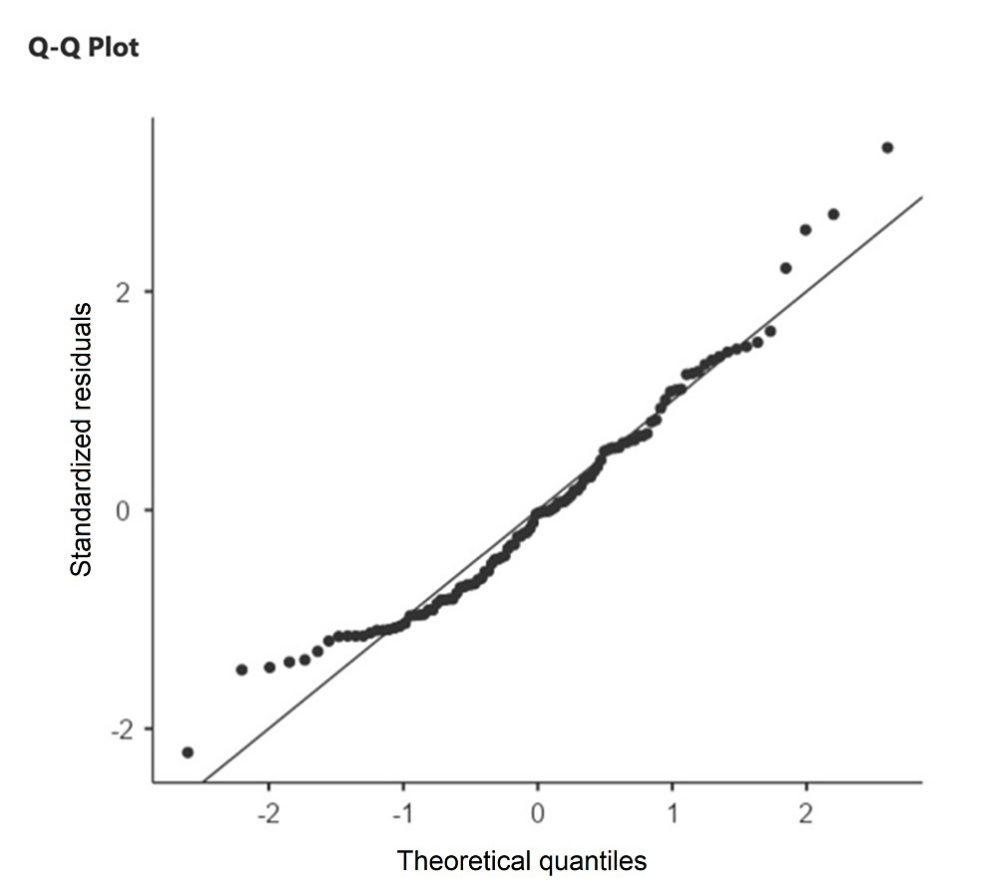
Quantile-quantile plot showing normality of data in the age groups of study population.

The majority of patients (71.2%, n=77) had random blood sugar levels between 150-200 mg/dL (Figure 4). We also observed that the admission rates in July (rainy season) were highest (19.4%) and the admission rates in December (winter season) were lowest (4%). The normality of data for different age groups was established by quantile-quantile plot, and analysis of the healing rates between different age groups was carried out through analysis of variance (ANOVA) (Figure 5). A significant variation was observed among the different age groups (p=0.045) (Table 2).

**Table 2:** Analysis of variance (ANOVA) for diabetic foot ulcer healing in different age groups. DF: degrees of freedom Here, p=0.045 shows significant difference in the healing rates between different age groups.

Source of variation	Sum of squares	DF	Mean square
Between groups	0.039	3	0.013
(Influence factor)			
Within groups	0.488	104	0.004
(Other fluctuations)			
Total	0.527	107	-
F-ratio	2.771	-	-

**Figure 6:**
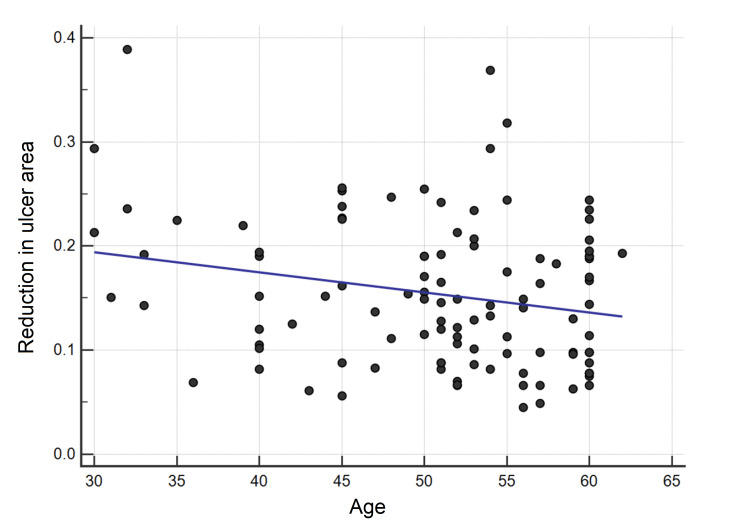
Linear regression curve showing relation between age and reduction in ulcer area (healing rate).

We observed significantly better healing rates for younger age groups as compared to older age groups. Post hoc test results showed that patients in age group of 30-39 years had better healing rate as compared to patients in age group of 50-59 years. Linear regression analysis was done with age as independent variable and reduction in ulcer area (healing rate) as dependent variable. It was seen that age was inversely proportional to the healing rate (p=0.019) (Figure 6).

**Figure 7:**
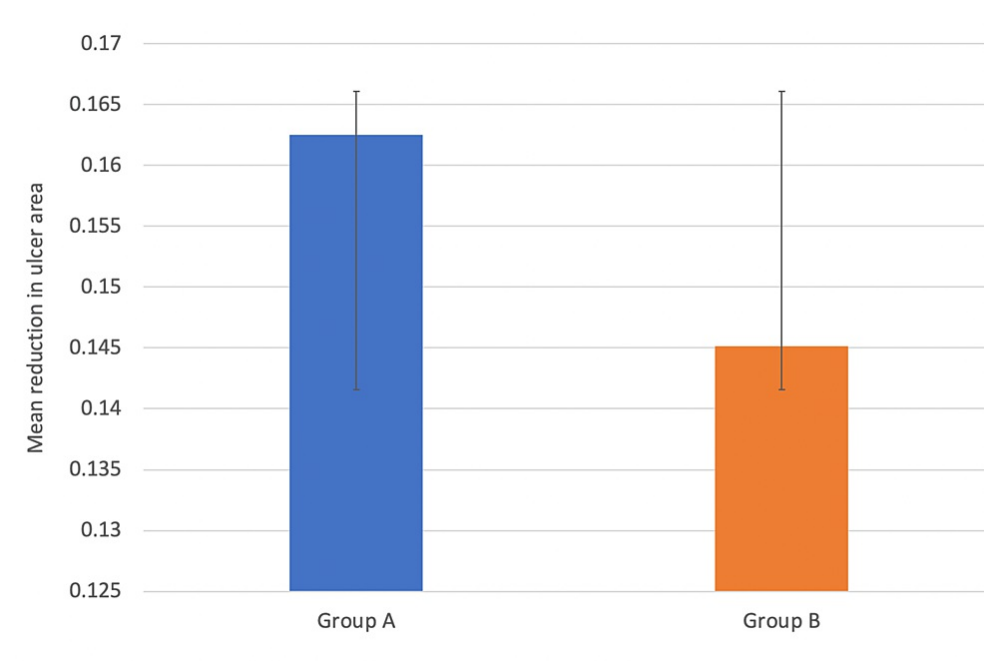
Bar graph showing comparison of means of healing rates of both groups with error bars showing standard deviation. Y-axis shows mean reduction in ulcer area. The means of group A and group B are compared using bar graph with error bars showing standard deviations.

Lastly, we compared healing rates of diabetic foot ulcer between group A (receiving topical sucralfate and mupirocin ointment) and group B (receiving topical mupirocin only) by calculating percentage reduction in ulcer area of each patient and then finding the mean values in both groups. The mean±SD of the percentage reduction in the wound area (healing rate) in group A was 16.2±7.3%, while in group B it was 14.5±6.6% as represented by bar graphs (Figure 7). Comparison of the mean values by Student's t-test failed to show a statistical difference in healing rates between the two groups (p=0.2015) (Table 3). The p-value was 0.201 which was statistically not significant, implying there was no statistically significant difference between the healing rates of the two groups.

**Table 3:** Comparison of mean healing rate in the two groups by Student's t-test. DF: degrees of freedom Here, the value of p=0.201 shows that there is no significant difference in the healing rates between the two groups.

Difference	-0.017
Standard error	0.013
95% CI	-0.044 to 0.009
Test statistic t	-1.285
DF	106
Significance level	P=0.201

## Discussion

Diabetic foot ulcer (DFU) is a common complication of diabetes. It affects up to 25% of diabetics in their lifetime [11]. These ulcers can lead to serious complications, including infections, amputations, and reduced quality of life. Topical sucralfate ointment has been proposed as a treatment for DFUs due to its ability to promote wound healing and reduce inflammation. To assess the effectiveness of this treatment, this study was conducted in the Department of Surgery, Rajendra Institute of Medical Sciences, Ranchi.

This study included both male and female patients, with a male-to-female ratio of around 3:1. Other studies have also observed that men were at a higher risk of developing foot ulceration in comparison to females [11,12]. The mean age at presentation in our study population was 51 years and majority of patients fell in the age group of 50-59 years. This was similar to a study by Akther et al., who found incidence of diabetic foot to be maximum in the age group of 41-60 years, with mean age of 55 years [13]. The healing rates between different age groups were analyzed using ANOVA, which showed a significant variation within the age groups (p=0.054). The study found that younger age groups had significantly better healing rates than older age groups. The admission rates in July, which is a rainy season in our part of India, were found to be the highest (19.4%), whereas the admission rates were the lowest in the winter month of December (4%). The majority of patients with diabetic foot in our study had diabetes for five to 10 years. This finding is consistent with the study of Boulton et al., which has shown that patients with longer duration of diabetes are at a higher risk of developing DFUs [14]. Almobarak et al. in their study observed a linear correlation between the duration of diabetes with the development of gangrene and ulceration [15]. Majority of the patients in our study had random blood sugar levels between 150-200 mg/dL. This was consistent with a study that also observed a correlation between poor glycemic control and diabetic foot lesions [16]. We calculated the healing rates by percentage reduction in ulcer area for each patient. The mean values of healing rates in both groups (group A and group B) were calculated and compared by Student's t-test. The mean±SD of the healing rates in the sucralfate group and the control group were estimated to be 16.2±7.3% and 14.5±6.6%, respectively, and comparison of the means by Student's t-test failed to show a statistical difference in healing rates between the two groups (p=0.201).

Thus, in our study, there was no statistical difference in the healing rates of diabetic foot ulcer with and without adding sucralfate to topical mupirocin ointment. This is in contrast with some studies, which have observed better healing rates of DFUs following the addition of sucralfate [17-20]. However, our study is consistent with the study by Ala et al., which did not observe any improvement in the healing rates of chronic ulcers after the addition of sucralfate [21].

Overall, our study provides valuable insights into the factors that influence healing rates in DFUs and highlights the need for further research to evaluate the effectiveness of sucralfate ointment in treating this condition. Further studies with larger sample sizes may be needed to determine whether sucralfate ointment has a statistically significant effect on healing rates in DFUs.

The limitation of our study was that it was a single-center study and the number of participants was less. Larger studies including participants from other centers would help us to arrive at a more decisive conclusion.

## Conclusions

Diabetic foot ulcer is a common and important complication encountered among patients with diabetes. Various topical treatment methods have been proposed for this disease. This study was conducted to evaluate the effectiveness of topical sucralfate and mupirocin combination over topical mupirocin alone for the treatment of diabetic foot ulcer. The study concluded that male sex had a greater risk of developing diabetic foot ulcers with maximum patients occurring in the age group of 50-59 years. Most patients had diabetes for five to 10 years. Younger patients had better healing rates. The incidence of diabetic foot ulcers peaked in the rainy season. There was no statistical difference in the healing rates of diabetic foot ulcers, between the topical sucralfate and mupirocin combination group and the topical mupirocin-only control group.

Thus, to conclude, the addition of topical sucralfate to mupirocin did not show any obvious benefits in terms of healing rates in diabetic foot ulcer treatment.
